# Flt3 Signaling in B Lymphocyte Development and Humoral Immunity

**DOI:** 10.3390/ijms23137289

**Published:** 2022-06-30

**Authors:** Kay L. Medina

**Affiliations:** Department of Immunology, Mayo Clinic, Rochester, MN 55905, USA; medina.kay@mayo.edu

**Keywords:** Fetal liver tyrosine kinase (Flt3), Flt3-ligand (FL), hematopoiesis, lymphopoiesis, B cells, signaling, survival, proliferation, cellular differentiation, transcriptional regulation

## Abstract

The Class III receptor tyrosine kinase Flt3 and its ligand, the Flt3-ligand (FL), play an integral role in regulating the proliferation, differentiation, and survival of multipotent hematopoietic and lymphoid progenitors from which B cell precursors derive in bone marrow (BM). More recently, essential roles for Flt3 signaling in the regulation of peripheral B cell development and affinity maturation have come to light. Experimental findings derived from a multitude of mouse models have reinforced the importance of molecular and cellular regulation of Flt3 and FL in lymphohematopoiesis and adaptive immunity. Here, we provide a comprehensive review of the current state of the knowledge regarding molecular and cellular regulation of Flt3/FL and the roles of Flt3 signaling in hematopoietic stem cell (HSC) activation, lymphoid development, BM B lymphopoiesis, and peripheral B cell development. Cumulatively, the literature has reinforced the importance of Flt3 signaling in B cell development and function. However, it has also identified gaps in the knowledge regarding Flt3-dependent developmental-stage specific gene regulatory circuits essential for steady-state B lymphopoiesis that will be the focus of future studies.

## 1. Introduction

**Figure 1:**
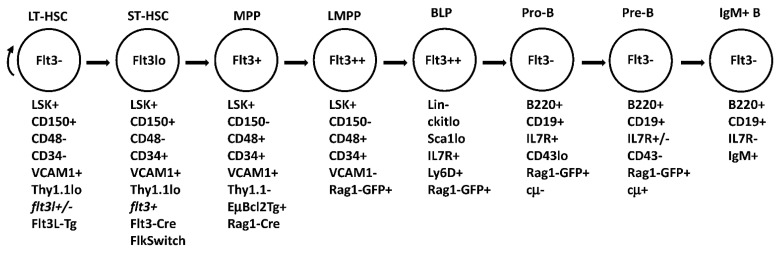
Developmental intermediates between HSC and IgM+ B cells. The phenotypic designations shown are integrated from mouse models discussed and referenced in the text. Flt3 expression shown in the circle represents protein. Italics indicate FL (flt3l) or Flt3 mRNA. B lineage progenitors (BLP) are a subset of CLP that have undergone B lineage specification. Cytoplasmic heavy chain protein is denoted cµ. Flt3L-Tg, Flt3-Cre, FlkSwitch, and Rag1-Cre are shown under the developmental stage in which transcript expression, indicative of promoter activation, has been detected.

Fms-like tyrosine kinase 3 (Flt3/Flk2) is a class III receptor tyrosine kinase that plays essential roles in normal and malignant hematopoiesis [1]. Flt3 is a membrane-bound cell surface receptor expressed at low levels on primitive hematopoietic progenitors in BM and is re-expressed on activated germinal center B cells [2,3]. Flt3-ligand, FL, is a type I transmembrane protein that upon cellular activation can be released as a soluble homodimeric protein [4]. FL is ubiquitously expressed, in hematopoietic and non-hematopoietic cells. Under steady-state conditions, serum levels of FL are low, as pre-formed FL is maintained in intracellular stores [4]. However, under stress conditions or upon cellular activation, intracellular stores of FL are transported to the cell surface and released as a soluble protein [5,6]. Flt3 signaling is largely held in check by the limited expression of Flt3 receptor. The critical roles of Flt3 signaling in lymphopoiesis, B cell development and maturation, and humoral immunity were revealed through the analysis of somatic receptor and ligand knockout mice that exhibit deficiencies in multipotent hematopoietic progenitors (MPP), common lymphoid progenitors (CLP), and B cell precursors in BM [7,8]. Transplantation studies revealed defects in lymphoid and myeloid reconstitution, but not HSC maintenance. Much effort has been put into understanding Flt3 signaling in hematopoietic development as mutations that render the receptor constitutively active are frequent in acute myeloid leukemia (AML) and confer a poor clinical outcome. Mutant Flt3 signaling in leukemogenesis has been the topic of many reviews and will not be discussed here. The focus of this review is to integrate the current state of the knowledge regarding molecular and cellular regulation of *flt3/*FL, the roles of Flt3 signaling in hematopoiesis, lymphoid and B cell fate specification and differentiation, and peripheral B cell development. We will review data obtained from a number of animal models (summarized in Table 1) and in vivo studies (summarized in Figure 1) that have provided clarification and new insight into Flt3 signaling, and that identify remaining gaps in the knowledge.

## 2. Molecular and Cellular Regulation of Flt3 and FL

Molecular and cellular regulation of *flt3* is tightly controlled and has been elucidated in the context of ex vivo isolated developmentally transitional BM progenitors as well as in a variety of hematopoietic cell lines. At present, eight transcription factors have been characterized as molecular regulators of *flt3*, including Myb, C/EBPα, Hoxa9, Meis1, Pbx1, PU.1, Bcl11a, and Pax5. DNaseI hypersensitivity assays revealed three cis-regulatory elements in the *flt3* genomic locus [25]. To identify key transcription factors as molecular regulators of *flt3* as a function of hematopoietic differentiation, BM hematopoietic progenitor subsets that differ in expression of Flt3 were purified, and DNaseI hypersensitivity assays were performed. The BM subsets evaluated included LSK+Flt3- enriched for HSC, LSK+Flt3+ enriched for MPP, and the myeloid/erythroid biased progenitor subsets CMP, GMP, and MEP that express low levels of Flt3 or are Flt3-. The *flt3* promoter was active in LSK+Flt3- HSC, LSK+Flt3+MPP, CMP, and GMP. Promoter occupancy in LSK+Flt3- HSC suggested that the *flt3* gene is primed for transcriptional activation in HSC. ChIP assays identified Hoxa9, Meis1, Pbx1, PU.1, Myb, and C/EBPα binding to the *flt3* promoter. Myb and C/EBPα were also bound to an intronic element that is functionally active only in LSK+Flt3+ MPPs. Importantly, neither promoter nor intronic nuclease sensitive sites were protected in MEP, consistent with the silencing of the *flt3* genomic locus upon commitment to the megakaryocyte and erythroid lineages. The functional significance of Myb and C/EBPα in regulation of *flt3* was confirmed by shRNA knockdown of Myb or C/EBPα that caused a reduction in *flt3* transcripts. Forced expression of Myb or C/EBPα in the HSC-like cell line HPC7 failed to induce Flt3; however, enforced co-expression Myb or C/EBPα resulted in upregulation of Flt3. These data indicate that Myb and C/EBPα functionally cooperate in the regulation of Flt3.

A similar strategy was used to determine the requirement for Hoxa9 in the regulation of Flt3 in lymphoid progenitors [26]. The cell surface expression of Flt3 is reduced in Flt3+ LSK+ and Flt3+ CLP in *hoxa9−/−* mice. ChIP assays revealed Hoxa9, Meis1, Pbx1, and PU.1 binding to the *flt3* promoter in Flt3+ multipotential *EBF1−/−* cells but not in Flt3- *RAG2−/−* Pro-B cells. The shRNA knockdown of Hoxa9 in Flt3+ *EBF1−/−* cells reduced *flt3* mRNA and protein. Importantly, the retroviral forced expression of Hoxa9 in the B lineage specified *Pax5−/−* cell line that expresses low levels of Flt3, upregulated *flt3* mRNA and protein. These experimental findings, together with those described above, implicate Hoxa9 in the regulation of Flt3 from the earliest stages of hematopoietic differentiation. Notably, *hoxa9−/−* and *FL−/−* mice share similar hematopoietic progenitor and B cell precursor deficiencies with respect to impaired Flt3 expression [21]. To determine if impaired lymphopoiesis and B cell genesis in *hoxa9−/−* mice was due to diminished Flt3 signaling, *FL−/−Hoxa9−/−* mice were generated [21]. Unexpectedly, the compound knockout mice exhibited a profound deficiency in lymphoid progenitors and an expansion of myeloid-biased HSC. These data suggest that Flt3 signaling synergizes with Hoxa9, from an extremely early checkpoint, to promote lymphoid developmental potential.

The transcription factor Bcl11a has also been implicated in the regulation of Flt3 in LSK+ progenitors and CLP. The frequencies of LSK+ and CLP were significantly reduced in *Bcl11a−/−* fetal liver cells [27]. Transplantation experiments confirmed that deficiencies in the frequencies of LSK+ or CLPs were cell intrinsic. Cre-ERT2 *Bcl11a^f//f/^* mice administered tamoxifen and analyzed 5–7 days post treatment revealed reductions in LSK+, LMPP, CLP, and B cell precursors. The qRT-PCR of LMPPs confirmed reductions in *flt3* transcripts. The Flt3+ *EBF1−/−* cell line was established from d14.5 fetal liver cells and would be an excellent tool to evaluate Bcl11a binding and functional relevance to *flt3* gene regulation [26].

As important as the induction of Flt3 expression in primitive progenitors is silencing Flt3 upon commitment to the B cell fate. Commitment and maintenance of the B cell fate is dependent on the transcription factor Pax5. Holmes, et al. identified Pax5 as a negative regulator of Flt3 in committed B cell progenitors [28]. Pax5 is bound to the *flt3* promoter in Pro-B cells. The authors further determined that Pax5 repression of *flt3* is critical for B cell development. Retroviral forced expression of Flt3 in BM HSC impaired B lymphopoiesis by decreasing B cell precursor frequency.

Molecular and cellular regulation of *flt3l* is also important for normal B lymphopoiesis and impacts expression levels of Flt3 [18]. The heterozygosity of *flt3l* reduced FL transcripts and serum levels. *FL+/-* mice had reductions in frequencies of LSK+Flt3+MPPs and reduced expression of Flt3 on LSK+Flt3+MPP. The reductions in LSK+Flt3+MPP in *FL+/-* mice coincided with reduced lymphoid priming, as indicated by impaired RAG1 locus activation, in addition to reduced frequencies of RAG1 expressing CLP and B cell progenitors. While this study established that threshold levels of FL are limiting for steady-state B lymphopoiesis, other reports support that the regulation of FL availability must be tightly regulated, independent of controlled expression of Flt3. FLT3L-Tg mice that constitutively express high levels of human FL, exhibit increased BM cellularity, expansion of myeloid cells, splenomegaly, and blood leukocytosis [20]. The FLT3L-Tg mice also developed anemia and platelet deficiency. The anemia and platelet deficiencies were reflected by a dramatic decrease in MEP with an increase in GMP, consistent with expanded myelopoiesis. Regarding BM B lymphopoiesis, flow cytometry analysis of FLT3L-Tg mice revealed expanded Ly6D+ CLP (shown as BLP in Figure 1), a normal Pro-B compartment, but reductions in Pre-B and IgM+ B cells. Interestingly, both e*bf1* and *pax5* transcript levels were reduced in FLT3L-Tg mice, suggesting that an overabundance of FL impairs the activation of the genetic circuitry that drives B cell commitment. Thus, while an over-abundance of FL expands the lymphoid progenitor pool, it impairs the differentiation of committed B cell precursors [24]. Similar results were obtained upon retroviral overexpression of Flt3 [28].

## 3. Flt3 Is Dispensable for HSC Generation and Maintenance

In mice, immunophenotyping studies coupled with functional assays and molecular analyses have established that the differential expression of Flt3 discriminates hematopoietic progenitor subsets. Flt3 is not expressed on HSC, consistent with in vivo studies that neither Flt3 nor FL are required for the steady-state hematopoiesis or the reconstitution of HSCs after primary or serial transplantation [9,29,30]. To further discriminate hematopoietic stem and progenitor cell functional subsets, the expression profiling of purified progenitors was performed. The analysis of HSC, CMP, and CLP identified VCAM1 as a marker expressed in HSC and CMP but not CLP. VCAM1+ Thy1.1lo Flt3- cells were enriched for HSC, the expression of Flt3 on VCAM1+ Thy1.1lo was distinguished HSC from short-term hematopoietic stem cells (ST-HSC), the loss of Thy1.1 on VCAM1+ Flt3+ cells distinguished MPP from HSC, and VCAM1- Flt3+ cells were enriched for lymphoid-biased multipotential progenitors (LMPP) that displayed higher surface levels of Flt3 and evidence of lymphoid priming [31,32]. These data show that differential expression of VCAM1 and Flt3 are effective markers to resolve multiple primitive hematopoietic progenitor subsets (Figure 1).

LSK+ subsets can also be fractionated based on the differential expression of CD150, CD48, CD34, and Flt3 (Figure 1). Only a small fraction of LSK+CD150+CD48- cells enriched for HSC express Flt3. Frequencies of Flt3+ cells are increased in LSK+CD150+CD48+ that co-express CD34. The upregulation of Flt3 within CD48+CD34+HSPC coincided with downregulated CD150 [33]. Another group performed single-cell analysis of *flt3* mRNA on progenitor subsets using the differential expressions of CD150 and CD48 [34]. A very low frequency of single LSK+CD150+CD48- HSC expressed *flt3* mRNA. Further flow cytometry analysis determined that within the LSK+CD150+CD48- subset, Flt3+ cells were largely restricted to progenitors displaying low levels of CD150 and expressing CD34. Notably, very little co-expression of *flt3* and *epor* mRNA was found in the same cells, consistent with the suppression of Flt3 in MEP.

Flt3-Cre^tg/+^R26R^EYFP/+^ reporter mice were established for fate mapping [10]. In these mice, the mouse *flt3* promoter drives the expression of EYFP. Therefore, all cells expressing Flt3 and their progeny, regardless of Flt3 expression, are marked by EYFP. Not surprisingly, all lymphoid and most myeloid progenitors were EYFP+. Importantly, all stages of MEP expressed EYFP, although they lacked Flt3 expression by flow cytometry. Consistent with functional studies demonstrating that Flt3 signaling is not required for HSC maintenance, most LSK + CD150 + CD48- lacked the expression of EYFP.

FlkSwitch reporter mice, also generated for fate mapping studies, further reinforced the finding that HSC maintenance is independent of Flt3 signaling [11]. Flk2Cre driven by Flk2 (Flt3) regulatory elements were crossed to mice expressing the dual fluorescent reporter mT/mG in the Rosa26 locus [35]. In this model, all cells express fluorescent membrane-targeted tandem dimer tomato (mT) prior to Cre excision and green fluorescent protein (GFP, mG) after Cre excision, allowing for the discrimination of recombined and non-recombined cells. All HSC (LSK + CD150 + CD48-Flt3-) expressed mT but not mG. ST-HSC discriminated by low levels of Flt3 were comprised of ~50% mG+, whereas >90% Flt3+ MPP were mG+, as well as CMP, CLP, MEP, GMP, and differentiated platelets and erythroid progenitors. Cre mRNA was evaluated in HSC, Flt3+ MPP, myeloid progenitors (Lin-ckit^hi^Sca1-), and erythroid progenitors (Ter119-CD71+). Notably, Cre expression and activity was lacking in cells that did not express and had downregulated or silenced Flt3. Not surprising, the floxing efficiency increased with Flt3 expression on hematopoietic progenitors. The FlkSwitch mouse substantiated that all hematopoietic lineages differentiate from a Flt3+ intermediary stage under homeostasis, after sublethal irradiation, or upon transplantation. This mouse model further provided definitive evidence that HSC specification and maintenance are Flt3-independent. The same group established that Flk2 expression does not restrict multilineage potential but allows for the isolation of highly functional HSC (mT+, Flk2-) [12]. A follow-up study by the lab using *FL−/−* and FlkSwitch reporter mice focused on the impact of Flt3 deficiency on myelopoiesis. In this study, they determined that Flk2 deficiency does not promote cell fate decisions but promotes cellular proliferation. An important observation from this study was that Flk2 deficiency provided a survival advantage after myeloablative stress due to reduced proliferation and cycling, which may have clinical applications [13]. Finally, the recently reported Flk2-Cre HSC-DTR mouse model that selectively expresses the diphtheria toxin receptor (DTR) in HSC will enable future studies of aspects of HSC biology that have remained elusive with existing models [15].

Signaling molecules that play essential roles in embryonic development may not precisely reflect adult hematopoiesis. Examination of fetal and neonatal progenitor subsets using the FlkSwitch reporter identified mT+ and mG+ fractions within the LSK + CD150 + CD48- subset enriched for HSC, suggesting the coexistence of two HSC subsets in early life [14]. Notably, both E14.5 fetal liver mT+ and mG+ were capable of long-term multilineage reconstitution after transplantation. The mT+ and mG+ HSC expressed comparable levels of CD34, but the mG+ expressed lower levels of CD150. The mG+ HSC represented a transient HSC population and were more proliferative as greater frequencies of mG+ were in S/G2/M with decreased frequency in G0/G1. The mG+ d14.5 FL HSC, although capable of long-term multilineage reconstitution, showed enhanced generation of lymphoid cells and reduced production of myeloid cells. Quantitative PCR showed lymphoid priming in mG+ compared to mT+ HSC. Interestingly, the mG+ FL HSC efficiently generated innate-like B cells in vivo, compared to mT+ FL HSC. The mG+ FL HSC also exhibited higher chimerism toward marginal zone B cells in the spleen than adult mT+ HSC.

## 4. Flt3 Signaling as a Driver of HSC Activation and Lymphopoiesis

Exit from quiescence is a hallmark of HSC activation [33]. *Flt3* and *flt3l* mRNA are enriched in HSC subsets, showing increased cell cycling [22]. BM hematopoietic progenitors maintain intracellular stores of pre-formed FL protein [6]. The activation of LSK+ cells by early acting cytokines (SCF, IL6, IL3, TPO) strongly induced surface expression of FL [6]. Thus, FL may promote hematopoietic progenitor proliferation, in part, through autocrine mechanisms.

Tornack, et al. generated an FL-GFP reporter mouse to elucidate cells within the BM microenvironment that express FL [22]. Interestingly, the FL-GFP reporter indicated that LSK + CD150 + CD48-Flt3-CD34- cells enriched for HSC could be subdivided into FL-GFPlo and FL-GFPhi subsets. The FL-GFPhi HSC had increased frequencies of cycling cells, while FL-GFPlo HSC had decreased frequencies of cycling cells. FL-GFPhi HSC expressed CD47, a marker of activated HSC [36]. Transplantation studies revealed that the FL-GFPlo HSC showed long-term reconstitution, while the FL-GFPhi HSC showed only short-term reconstitution.

Flt3/FL signaling rapidly and in a FL dose-dependent manner induces the phosphorylation of AKT and the forkhead transcription factor FOXO3 [37]. The phosphorylation of FOXO3 results in inactivation and nuclear export. Activated FOXO3 is essential for the maintenance of the HSC pool as *foxo3a−/−* HSC exhibits defective maintenance of quiescence [38]. FOXO3 regulates the proliferation via the transcriptional regulation of the cyclin-dependent kinase inhibitor p27kip1 and cyclin D. The suppression of FL may conserve HSC quiescence by inhibiting proliferation. The upregulation of Flt3, coupled with the release of intracellular stores of FL, may be an important hub in a signaling network, leading to the inactivation of FOXO3, promoting HSC activation and FL-promoting proliferation. FOXO3 also contributes to progenitor survival via the transcriptional regulation of pro-apoptotic gene promoters, including Bim and FasL. Constitutively active FOXO3 induces Bim and p27kip1 even in the presence of Flt3 signaling. Thus, FOXO3 phosphorylation may also represent a primary target for FL mediated regulation of hematopoietic stem and progenitor survival [37].

Flt3 signal transduction cascades initiated by FL binding induce the autophosphorylation of tyrosine residues. Tyrosine autophosphorylation creates docking sites for the p85 subunit of PI3K, Ras GTPase, phospholipase C-γ, Shc, Grb2, and Src (reviewed in [39]). Mice harboring a dominant negative form of Ras (dnRas) exhibit defective B lymphopoiesis at the CLP to Pro-B stage, similar to *flt3−/−* mice [40]. Both *flt3−/−* and dnRas display reduced IL-7R expression and impaired proliferation but not survival. dnRas mice crossed to STAT5b-CA mice did not show restoration of CLP, although numbers of Pro-B cells were restored. The rescue was not due to increased proliferation. The dnRas CLP and B cell precursors showed increased *socs2* and *socs3,* which were suggested to reduce Stat5 activation downstream of IL-7 signaling. These data suggest that Flt3 signaling via Ras primes the ability of CLP and B cell progenitors to respond to IL-7 and activate Stat5.

## 5. Flt3 Signaling in B Lymphopoiesis

Previous studies confirmed critical roles for FL and IL-7 in B cell development as manifested by the ablation of B lymphopoiesis in *FL−/−IL7−/−* compound knockout mice [23]. However, the role of Flt3 signaling alone in lymphoid/B cell development was less clear. Mice deficient in Flt3 signaling exhibit reduced lymphoid priming and impaired B lymphopoiesis, supporting a nonredundant role for Flt3 signaling in the generation of lymphoid lineage progenitors that give rise to committed B cell precursors [6]. To determine if Flt3 signaling could instruct B cell commitment in the absence of IL-7, FLT3L-Tg mice were assessed for ability to rescue the B cell deficiency in *IL-7−/−* mice [24]. While numbers of CD19+ Pro-B cells were rescued in FLT3L-Tg *IL7−/−* mice, Pre-B cells and immature CD19 + IgM+ B cells were not rescued to wildtype levels. The CD19+ Pro-B cells generated in the FLT3L-Tg *IL7−/−* mice expressed reduced but permissive levels of EBF1, Pax5, and FOXO1 to mediate B cell commitment. This experimental finding suggests that Flt3 signaling is permissive and sufficient for the expansion of Pro-B cells and that a weak induction of essential transcriptional regulators is required for B cell commitment but cannot bypass the requirement for IL-7 signaling in B cell differentiation after lineage commitment.

Signaling molecules also contribute to cellular differentiation programs by regulating survival pathways. *FL−/−* RAG1GFP+/− EμBcl2Tg mice were established to examine the role of Flt3 signaling in the survival of lymphohematopoietic progenitors and B cell precursors in BM [19]. EμBcl2Tg expression in *FL−/−* RAG1GFP+/− mice increased frequencies and numbers of LSK+ cells but did not significantly alter the frequencies of LMPP and had no impact on lymphoid priming, as determined by RAG1GFP expression. Similar findings with *FL−/−* EμBcl2Tg mice without the RAG1-GFP reporter were obtained by von Muenchow, et al., noting partial restoration of Flt3+ CLPs and CD19+ Pro-B cells [24].

Flt3^fl/fl^ Vav1-Cre were generated to investigate the role of Flt3 in lymphoid development, prior to lymphoid specification [16]. Consistent with the findings detailed above, the pan-hematopoietic loss of Flt3 had no effect on HSC. Reductions in LSK + CD150-CD48 + Flt3+ MPP were observed, as this subset includes the majority of MPPs. CLPs were also reduced, consistent with normal expression of Flt3. Flt3^fl/fl^ Mx1-Cre showed identical results regarding HSC. In contrast to Flt3^fl/fl^ Vav1-Cre, Flt3^fl/fl^ Mx1-Cre showed no effect on CLP, suggesting that CLP maintenance is not as dependent on Flt3. Interestingly, a genomic analysis of BM B lineage precursors revealed that the majority of residual CLP retained a non-deleted floxed allele, suggesting a competitive advantage of Flt3+ progenitors over Flt3- in sustaining steady-state B lymphopoiesis. Finally, Rag1-Cre was used to target the loss of Flt3 downstream of lymphoid priming. There was no change in the Flt3 expression on LMPP, but there was a significant reduction in Flt3+ CLPs in Flt3^fl/fl^ Rag1-Cre mice. The reduction in Flt3+ CLP led to corresponding reductions in Pro-B, Pre-B, and IgM+ B cells. These data reinforce a strict requirement for Flt3 in lymphoid progenitors after lymphoid priming. This was similarly true for fetal B lymphopoiesis. Taken together, the data obtained from the loss of Flt3 in LSK+ progenitors or after initiation of lymphoid priming reinforce a non-redundant role for Flt3 signaling in the regulation of the MPP pool and lymphoid/B cell development.

## 6. Regulation of Peripheral B Development by Flt3/FL

FL plays an integral role in steady-state and stress hematopoiesis. The observation that serum levels of FL increase dramatically in BM-failure-associated blood disorders, suggesting that under physiologic conditions of stress, hematopoiesis release of intracellular stores of FL is a mechanism to restore normal hematopoiesis [41,42]. Subsequent studies focused on the cellular source of FL and found that T cells were a major source of pre-formed FL in the periphery [5]. It was subsequently determined that gamma-chain-sharing cytokines are efficient inducers of membrane-bound and soluble FL from human T cells, providing insight into the mechanism of T cell release [43]. A link to humoral immunity was provided when it was observed that exogenous FL differentially impacted cytokine production by antigen-specific T cells in vivo and enhanced antibody titers in blood [44]. Interestingly, the antibody isotype was biased to IgG2a compared to IgG1 and was the result of higher frequencies of antibody-producing cells. This finding was further corroborated with it was shown that patients with Sjogren’s Syndrome showed increased serum levels of FL and increased frequency of Flt3+ B cells in peripheral blood. Notably, exogenous FL enhanced anti-IgM mediated proliferation and survival in vitro. Blocking FL or Flt3 impeded patient survival [45]. Studies were then focused on understanding the mechanisms by which Flt3 signaling impacts humoral immunity. Flt3 was found to be re-expressed on activated germinal center B cells, and signaling activates a molecular circuitry critical for class switch recombination [17,46]. Together, these studies establish a crucial role for Flt3/FL signaling in the regulation of humoral immunity.

Flt3 signaling also impacts peripheral B cell maturation and homeostasis. *FL−/−* mice have reduced splenic cellularity, fewer transitional B cells, skewed frequencies of marginal zone B cells, and reductions in numbers of follicular B cells [47]. Bone marrow chimeras established with wildtype and *FL−/−* bone marrow cells normalized deficiencies in numbers of transitional and follicular B cells and the marginal zone B skewing. These results were corroborated when *FL−/−* mice were administered exogenous FL, establishing that the peripheral B cell maturation alteration was cell-extrinsic. An additional role for Flt3 in peripheral B cell homeostasis was suggested in results obtained from mixed radiation chimeras transplanted with fetal liver cells from Flt3^fl/fl^ Rag1-Cre and wildtype competitor cells. Eight weeks post-transplant the authors found impaired reconstitution of B1a, marginal zone B, and conventional B cells in the peritoneal cavity and spleen of the transplant recipients [16]. Thus, in addition to being a critical regulator of BM B lymphopoiesis, Flt3 signaling plays roles in peripheral B cell maturation and class switch recombination.

## 7. Conclusions and Future Directions

Here, we summarized the current experimental findings that substantiate and reinforce the nonredundant role of Flt3 signaling in lymphohematopoiesis and B cell development. Impaired Flt3 signaling, due to deficiencies in Flt3 or FL, dysregulated expression of Flt3, or overproduction of FL, disrupts B cell development in BM, as well as peripheral B cell differentiation and homeostasis. At present, seven transcription factors have been implicated in the regulation of *flt3*, and additional experimental models are needed to investigate their combinatorial roles with Flt3 signaling in the regulation of B lymphopoiesis, similar to Hoxa9. A major gap in the knowledge is how Flt3 signaling can bypass the requirement for IL-7 signaling in the activation of the transcriptional circuitry driving commitment to the B cell fate. The molecular mechanism by which Flt3 signaling suppresses megakaryocyte/erythroid development remains unclear. Signaling molecules can impact cell-fate decisions by altering the expression or activity of key lineage determining transcription factors. Additional studies, at the single-cell level, are needed to determine if Flt3 signaling alters the balance of transcription factors that promote MEP or B cell differentiation and the precise stages impacted. The experimental findings in this review were confined to data obtained in mouse models or murine cell lines. We know very little concerning the role of Flt3 signaling in human hematopoiesis and B cell development. The latter is particularly important given the impact of FL dysregulation in human disease.

## Figures and Tables

**Table 1:** Mouse models to study Flt3/FL signaling in hematopoiesis and humoral immunity.

Mouse Strain	BM and/or Peripheral B Cell Phenotypes	Functional Application	References
Flt3/Flk2-/-	Reductions in CLP and diminished frequencies of all BM B lineage cells. No reductions in HSC.	Determine the requirement for Flt3 in hematopoiesis, HSC maintenance in steady-state and upon hematopoietic stress.	[7,9]
Flt3-Cre^tg/+^ R26R^EYFP/+^	All HSC progeny, but not HSC, express EYFP.	Fate mapping. The *flt3* promoter drives EYFP; all cells expressing Flt3 and their progeny are marked by EYFP expression.	[10]
FlkSwitch	All HSC progeny, but not HSC, express GFP. Identification of fetal HSC with increased capacity to generate innate-like B and T cells. FL deficiency protects against myeloablative insult.	Fate mapping driven by Flk2Cre excision. All cells express membrane Tomato (mT) prior to Cre expression. Flk2 activated Cre excises mT and all recombined progeny are labeled GFP+.	[11,12,13,14]
Flk2-Cre HSC-DTR	HSC specific cell death induced by administration of diphtheria toxin (DT)	Selective investigation of HSC biology under varying physiologic conditions.	[15]
Flt3^fl/fl^ Vav1-Cre	Flt3 signaling not required for establishment or maintenance of HSC in fetal or adult hematopoiesis.	Panhematopoietic loss of Flt3 in all hematopoietic cells.	[16]
Flt3^fl/fl^ Mx1-Cre	Reductions in Flt3+ MPP but no reductions in CLP suggesting Flt3 nonredundant for maintenance of CLP.	Hematopoietic loss of Flt3 in HSC and differentiated progeny.	[16]
Flt3^fl/fl^ Rag1-Cre	Reductions in frequencies of Flt3+ CLP; reductions in Pro-B, Pre-B, and IgM+ B cells in BM, suggesting requirement for Flt3 signaling after initiation of lymphoid gene expression.	Requirement for Flt3 signaling downstream of lymphoid priming in B cell development. Also used to evaluate requirement for Flt3 signaling in fetal hematopoiesis.	[16]
Flt3l (FL)-/-	Reduced BM cellularity, numbers of Flt3+ MPP, CLP, BCP. Reduced spleen cellularity, reductions in numbers TS and FO, but not MZB cells. Defect in class switch recombination.	Determine the requirement for FL in the development of hematopoietic progenitors in BM and B cell maturation in the periphery.	[8,9,17]
FL-/- Rag1GFP	Reduced GFP+ LSK+, GFP+ CLP, and GFP+ BCP.	Lymphoid/B lineage tracing and priming.	[18]
FL+/- Rag1GFP	Reductions in *flt3l* mRNA and serum FL. Mice have reductions in GFP+ LSK+, GFP+ CLP, and GFP+ BCP.	Determination of dose-dependent requirement of FL in hematopoietic and lymphoid development.	[18]
FL-/- Rag1GFP EmBcl2Tg	Increased MPP, CLP and Pro-B cells. No increase in lymphoid priming in LSK+ or CLP.	Impact of FL deficiency on the survival of lymphoid/B cell progenitors.	[19]
FLT3L-Tg	Blood leukocytosis, splenomegaly, anemia, and reductions in platelets. Increase in BM lymphoid and myeloid progenitors. Reductions in PreB and IgM+ B cells in BM.	Determine the effect of FL overproduction on BM hematopoiesis and B cell differentiation.	[20]
FL-/-Hoxa9-/-	Expanded frequencies in myeloid-biased HSC and more severe reductions in Flt3+ MPP, BLP and B cell subsets than in FL-/- or Hoxa9-/- single knockouts.	Determine the combinatorial roles of Hoxa9 and Flt3 signaling in regulation lymphoid/B cell development.	[21]
FL-GFP	Discrimination of FL-GFPlo LT-HSC from FL-GFPhi proliferating ST-HSC.	Identification and characterization of hematopoietic and non-hematopoietic sources of FL.	[22]
FL-/-IL7-/-	Complete ablation of BM and fetal liver derived B cells. No detectable serum immunoglobulin.	Determination of single versus complementary roles of FL and IL-7 in lymphocyte development.	[23]
Flt3L-Tg IL7-/-	FL signaling is permissive for B cell survival and commitment but not IL-7 directed differentiation of Pro-B cells.	Determination if FL can bypass the requirement for IL-7 signaling in B cell development.	[24]

## Abbreviations

FL	Flt3-ligand
LT-HSC	long-term hematopoietic stem cell
ST-HSC	short-term hematopoietic stem cell
LSK+	Lineage-negative Sca-1+ ckit+
CMP	common myeloid progenitor
CLP	common lymphoid progenitor
GMP	granulocyte/monocyte progenitor
MEP	megakaryocyte/erythroid progenitor
LMPP	lymphoid-biased multipotential progenitor
BM	bone marrow
